# Hospital Frailty Risk Score Is Independently Associated with Mortality and Encephalopathy in Hospitalized Patients with Hepatocellular Carcinoma

**DOI:** 10.3390/biomedicines9111693

**Published:** 2021-11-15

**Authors:** Daryl Ramai, Khoi P. Dang-Ho, Anjali Kewalramani, Praneeth Bandaru, Rodolfo Sacco, Luca Giacomelli, Aashni Shah, Simonetta Papa, Francesca Cappellini, Fabio Perversi, Sara di Nunzio, Antonio Facciorusso

**Affiliations:** 1 Division of Gastroenterology and Hepatology, University of Utah, Salt Lake City, UT 84113, USA; Daryl.Ramai@hsc.utah.edu; 2 Department of Internal Medicine, The Brooklyn Hospital Center, Brooklyn, NY 11201, USA; kpdangho@tbh.org; 3 Department of Internal Medicine, Mathers Hospital, Port Jefferson, NY 11777, USA; kewalramanian@gmail.com; 4 Division of Gastroenterology and Hepatology, The Brooklyn Hospital Center, Brooklyn, NY 11201, USA; pbandaru@tbh.org; 5 Section of Gastroenterology, Department of Medical and Surgical Sciences, University of Foggia, 71122 Foggia, Italy; r.sacco@ao-pisa.toscana.it (R.S.); antonio.facciorusso@virgilio.it (A.F.); 6 Polistudium SRL, 20135 Milan, Italy; aashni.shah@polistudium.it (A.S.); simonetta.papa@polistudium.it (S.P.); Francesca.Cappellini@polistudium.it (F.C.); Fabio.Perversi@polistudium.it (F.P.); sara.dinunzio@polistudium.it (S.d.N.)

**Keywords:** hepatocellular carcinoma, frailty, outcomes

## Abstract

Frailty represents a state of vulnerability to multiple internal physiologic factors, as well as external pressures, and has been associated with clinical outcomes. We aim to understand the impact of frailty on patients admitted with hepatocellular carcinoma (HCC) by using the validated Hospital Frailty Risk Score, which is implemented in several hospitals worldwide. We conducted a nation-wide retrospective cohort study to determine the effect of frailty on the risk of in-patient mortality, hepatic encephalopathy, length of stay and cost. Frailty was associated with a 4.5-fold increased risk of mortality and a 2.3-fold increased risk of hepatic encephalopathy. Adjusted Cox regression showed that frailty was correlated with increased risk of in-patient mortality (hazard ratio: 2.3, 95% CI 1.9–2.8, *p* < 0.001). Frail HCC patients had longer hospital stay (median 5 days) vs. non-frail HCC patients (median 3 days). Additionally, frail patients had higher total costs of hospitalization ($40,875) compared with non-frail patients ($31,667). Frailty is an independent predictor of hepatic encephalopathy and in-patient mortality. Frailty is a surrogate marker of hospital length of stay and cost.

## 1. Introduction

Hepatocellular carcinoma (HCC) is the fourth most common type of cancer in the world and is a leading cause of cancer-related mortality, primarily due to untreated hepatitis C virus, alcoholic hepatitis, and nonalcoholic steatohepatitis [1]. HCC is increasingly adding to the burden of cancer-related mortality with a 5-year survival of <12% [1]. With advances in surgical techniques, surgery offers a potential cure for HCC; however, it remains controversial in the elderly population. It is suggested that after liver resection, there is a delay in the regeneration of the liver remnant due to the liver’s decreased proliferative capacity [2,3]. Indeed, normal aging is associated with a gradual decline in liver volume, blood flow to the liver and metabolic and synthetic function [2,3].

Frail adults can have alterations in their glucose metabolism, dysregulation of the autonomic nervous system, and age-related changes in the renin–angiotensin system and mitochondrial function [4]. Inflammatory markers, including IL-6, may also play a direct role in contributing to frailty [5]. Sarcopenia is a likely contributing factor in frailty due to loss of muscle mass and strength. Sarcopenia is reported in approximately one-third of patients with HCC and is an independent predictor of mortality [6]. 

Further studies have demonstrated that dysregulation of the stress response systems play an important role in the development of frailty. To this end, the dysregulation of stress response is driven by environmental changes, genetics, and age-related molecular changes [6].

While the number of elderly patients with HCC is expected to rise, their management is more complicated due to the presence of comorbid conditions [7,8]. However, age alone is insufficient to properly assess risk–benefit tradeoffs in HCC [9]. Treatment patterns vary amongst providers and some patients may be over- or under-treated, as there is a lack of consensus between existing risk assessment tools to identify elderly patients with increased risk of toxicity and adverse outcomes [1]. Additionally, generalizing the results obtained from phase III clinical trials to the elderly population may pose a risk on those who are frail and vulnerable and lead to increased morbidity and mortality [1].

Indeed, to assess physiological reserve and functional capacity, a more thorough measure is needed to determine overall risk. To this end, frailty should be regarded as vulnerability to multiple internal physiologic factors, as well as external stressors. Frailty describes a physiological decline in function due to aging which includes the inability to adapt and respond to stressors [10]. This process may occur at varying rates in different individuals [10].

Frailty increases the risk of other geriatric pathologies and adverse health outcomes [11]. The frailty phenotype is the most frequently used screening tool [12]. However, an assessment of the potential use of frailty as an index of measurement of healthcare-related outcomes in patients with HCC is lacking.

The approach to measuring frailty is based on the accumulation of illness, cognitive decline, and social illness [13]. Frailty syndrome is defined as meeting at least 3 of the following criteria: slowed walking speed, weakness as measured by low grip strength, unintentional weight loss, self-reported exhaustion, and low level of physical activity [13]. Another well-established tool is the comprehensive geriatric assessment, which looks at factors including nutritional status, mental and cognitive status, and physical medical comorbidities by utilizing laboratory values and assessment tools [14].

The Hospital Frailty Risk score provides a cost-effective, systematic way to screen patients for frailty to aid in prognostication and allocation of medical resources [15]. This scoring system has the advantage of using the International Statistical Classification of Diseases and Related Health Problems, Tenth Revision (ICD-10) codes. This allows for implementation within hospital information systems utilizing ICD-10 coding systems. The scoring system also accounts for variations in coding or the depth of coding and is predictive of poor outcomes regardless of coding depth.

We aim to understand and apply the Hospital Frailty Risk Score [15], a validated index, in patients admitted with HCC to assess the risk of inpatient mortality and hepatic encephalopathy. Hepatic encephalopathy was studied as a significant proportion of patients with chronic liver disease and malignancy are at increased risk. We also evaluated secondary outcomes including cost and hospital length of stay.

## 2. Materials and Methods

### 2.1. Data Source

A retrospective cohort study was conducted using the Healthcare Cost and Utilization Project (HCUP) Nationwide Inpatient Sample (NIS), USA, for 2014 [16]. NIS is a database of inpatient admissions and represents about 20% of US non-federal acute care hospitals. Using International Classification of Diseases 9th Revision (ICD-9-CM) codes, we extracted patients admitted with HCC. Demographical variables (age, gender, and ethnicity), length of hospital stay, elective admission, hospital teaching status, number of beds, type of insurance (Medicare, Medicaid, private, self-pay, other), discharge outcome (death or survival), hepatic encephalopathy, obesity, and depression were extracted. 

### 2.2. Exposure

We calculated the Hospital Frailty Risk Score at the time of hospitalization for patients admitted with a diagnosis of HCC. Frailty index was created and endorsed using 1 million patients who were over the age of 74 years [15]. We used this frailty score to identify patients who were at higher risk of healthcare-related outcomes, including mortality, hepatic encephalopathy, and length of stay. We created separate categories, low and high risk to aid in the interpretation of our results with cut-off points to distinguish outcomes between different individuals. Patients were determined to be of low frailty risk with a score of <5, while patients of medium frailty risk had a score of 5–15, and high frailty risk with a score of >15. Since the high frailty group accounted for only approximately 8% of the total cohort, we merged medium and high frailty patients into one group.

### 2.3. Characteristics

Baseline patient characteristics included age, sex, race or ethnicity, type of insurance (Medicaid, private insurance, self-pay, and other insurance types), and relevant comorbidities used to determine the Charlson comorbidity index. In addition, patient data included transplant surgery, depression, obesity, and depression. For each hospitalization, we captured admission (elective or non-elective), number of beds (small, medium, or large), hospital teaching status (rural, urban, or urban teaching), and length of hospital stay (defined as less than or greater than 7 days).

### 2.4. Outcomes

Our primary outcomes were the risk of inpatient mortality and the development of hepatic encephalopathy. Secondary outcomes included hospital length of stay and total costs of hospitalization. Total cost was provided per individual patient within the National Impatient Sample (NIS) database and extracted for analysis. We calculated median and interquartile costs for frail and non-frail patients.

### 2.5. Statistical Analysis

Data were analyzed by descriptive statistics. Categorical values were represented as percentages while continuous variables were represented by median and interquartile range. Pearson χ^2^ tests were used to compare categorical variables. We used Kaplan–Meier estimations to compare survival rates between both cohorts. We used multivariable regression analysis to evaluate the effect of independent variables and frailty on mortality and/or hepatic encephalopathy outcomes. Cox proportion regression analysis was used to determine predictors of mortality outcomes. All statistical analyses were performed with SPSS Statistics (IBM, Version 25). The study was determined to be exempt from Institutional Review Board review because human subjects were not involved. Data were acquired from a de-identified registry publicly accessible.

## 3. Results

In total, 10,983 patients with HCC hospitalized in the year 2014 were identified. Of them, 2986 (27.2%) were females and the median age was 62 years (standard deviation ± 15.1 years). Most patients were Caucasian (54.5%). Most admissions occurred at large hospitals (61.5%) and more frequently at Urban teaching institutions (78.0%). Most patients had less than 7 days of inpatient stay (71.9%). Based on the Hospital Frailty Risk Score, 6335 (57.7%) patients were considered frail and 4648 (42.3%) were considered non-frail at index admission (Table 1).

Adjusting for age, ethnicity, sex, elective versus non-elective admission, transplant, depression, obesity, alcohol use, number of beds, teaching status, payer insurance, and hospital length of stay, frailty was independently correlated with a 4.5-fold increased risk of mortality (odds ratio (OR): 4.5; 95% confidence interval (CI): 3.7–5.5, *p* ≤ 0.001) (Table 2). In addition, frailty was associated with a 2.3-fold higher risk of hepatic encephalopathy (95% CI: 1.6–3.1, *p* < 0.001) (Table 3).

**Figure 1:**
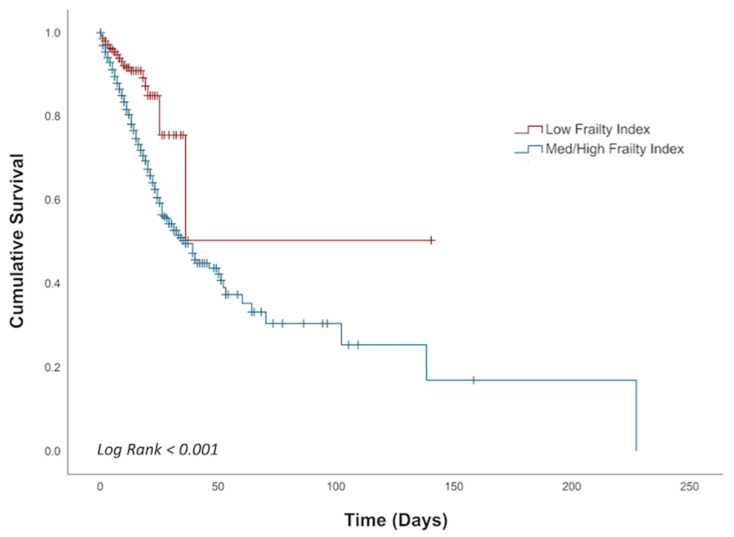
Kaplan–Meier estimation in determining the impact of frailty on in-patient mortality.

We performed Kaplan–Meier estimations to determine the impact of frailty on mortality. Kaplan–Meier estimations showed that higher frailty was associated with lower survival rates (log rank *p* < 0.001) (Figure 1). Adjusted Cox regression demonstrated that frailty was correlated with increased risk of in-patient mortality (hazard ratio (HR) 2.3, 95% CI: 1.9–2.8, *p* < 0.001). Patients who developed hepatic encephalopathy were also at higher risk for mortality during hospitalization (HR 2.2, 95% CI: 1.7–2.8, *p* < 0.001), as well as patients with increasing age 40–64 years (HR 5.5, 95% CI: 2.7–11.1, *p* < 0.001) and over 65 years (HR 5.7, 95% CI: 2.8–11.6, *p* < 0.001) (Table 4).

Frail patients with HCC were more likely to have longer hospital length of stay (median 5 days; interquartile range, 6 days) vs. non-frail patients (median 3 days non-frail patients; interquartile range, 3 days). Additionally, frail patients tend to have higher median total costs of hospitalization ($40,875; interquartile range, $60,764) compared to non-frail patients $31,667; interquartile range, $44,150).

## 4. Discussion

In this nationally representative study, representing about 20% of non-federal acute care hospitals in the US, we made several prognostic observations regarding the use of a validated hospital frailty index in patients with HCC. Firstly, frailty score was an independent predictor of mortality and hepatic encephalopathy in adjusted models. Secondly, frail patients were observed to have longer hospital stays and higher cost burden. Although the score serves as a good risk-stratification tool at a group level, individual outcomes are unpredictable in acute care settings.

The ability to use routine data for patients admitted to the hospital serves as an advantage of using hospital administrative data to determine frailty risk. Studies performed in the UK have employed the use of electronic medical records to develop an electronic frailty index, which is utilized in UK general practices [17]. Using frailty to evaluate treatment measures are recommended by international guidelines. The electronic frailty index was calculated by the presence or absence of individual deficits. It was used to categorize patients with mild, moderate, and severe frailty with strong predictive value for outcomes of mortality, nursing home admission, and hospitalization [17].

Frailty has been associated with the elevation of pro-inflammatory cytokines which is independently linked to increased morbidity and mortality [3]. Upregulation of cytokines, including IL-6, IL-10, and TNF-α, have been associated with poor mobility and functional status. In a study performed by Epps et al., mean IL-6 levels in frail patients was approximately three-times that of those found in non-frail patients [3]. Frailty has also been associated with superoxide anion overproduction by NADPH oxidase [18]. The Framingham Offspring study suggests oxidative stress as an underlying mechanism of frailty [18].

HCC develops as a result of chronic insults from HBC or HCV infection, chronic alcohol abuse, non-alcoholic steatosis, or exposure to aflatoxin B. The mediators of inflammation play an important role in the progression of HCC [19]. In the setting of HBC or HCV, hepatic oxidative stress is linked to increased risk of HCC. The chronic inflammation in HCC causes dysregulation of cellular pathways leading to cirrhosis and hepatocarcinogenesis [20].

Factors that contribute to general frailty, including altered gut microbiota, sarcopenia and endotoxemia, are drivers of liver decompensation in HCC [21]. Several factors contribute to sarcopenia in cirrhosis including low glycogen deposits, lack of adequate energy sources, and physical inactivity [21]. Alterations in the gut microbiota can lead to systemic endotoxemia which activates Toll-like receptors on muscle. This leads to increased protein breakdown and reduction in protein synthesis, resulting in sarcopenia. Sarcopenia, which is a key contributing factor in frailty and has been associated with a poor quality of life, mortality pre- and post-liver transplant, and longer hospital stay [21]. 

Patients with cancer are more likely to be frail and at higher risk for treatment-related morbidity and mortality [22]. Hurria et al. reported a multicenter prospective study that included multiple cancer types from 500 patients in the US [23]. The study showed a significant correlation between geriatric assessment variables and grade 3–5 toxicities. A meta-analysis concluded that the risk of morbidity was 2.7-fold higher in the elderly population when compared to younger patients with colorectal and liver metastases. However, the morbidity and mortality rates did not differ considerably between the younger and older population undergoing hepatic resection in the setting of HCC [24]. Another meta-analysis revealed a higher risk of postoperative renal failure, infection occurrence, and mortality in frail patients compared to non-frail undergoing liver resection [25]. 

Similarly, Aparicio et al. observed that patients with metastatic colorectal cancer starting first-line chemotherapy were associated with frailty-related factors, such as Instrumental Activities of Daily [26]. Another study by Gani et al. reported that frailty was predictive of poor outcomes following liver surgery [27]. Fong et al. concluded that age was an independent risk factor for morbidity in patients older than 65 years undergoing liver resection in the setting of colorectal liver metastasis [10]. A study conducted by Yamada et al. investigated the role of frailty in the prognosis of elderly patients undergoing hepatectomy in the setting of hepatocellular carcinoma. The frailty group included patients with larger tumor size, higher rate of postoperative complications, and longer length of stay. The study also showed worse disease-free survival rates in the frailty group [21]. 

Recently, Soto et al. reported that frail patients with liver cirrhosis were likely to have higher mortality rates at long-term follow-up [28]. A reduction in gait speed was highly associated with lower mortality and was proposed as a clinical indicator of frailty. In a study performed by Lai et al., Liver Frailty Index (LFI) scores were calculated and the association between LFI scores, hepatic encephalopathy, and mortality was investigated [29]. It was found that a larger number of frail patients with hepatic encephalopathy and ascites died who were on the liver transplant waitlist, compared to patients who were not frail. Frailty was determined to be a prevailing complication of cirrhosis and an independent risk factor for waitlist mortality [29]. Furthermore, frailty likely represents the end result of chronic hepatic synthetic dysfunction with muscle wasting and malnutrition, which plays a role in worsening overt portal hypertensive complications, including hepatic encephalopathy and, ultimately, mortality.

Sarcopenia has been associated with a high risk of hepatic encephalopathy likely due to decreased removal of ammonia from muscle in the setting of increased muscle breakdown in the elderly. Sarcopenia has also been identified as a predictor of hepatic decompensation [30].

As a result, frailty can be used to prognosticate and stratify patients with HCC. Furthermore, using this risk assessment, programs involving exercise, nutrition, and neurocognitive strategies may improve functional capacity and frailty scores, with better results when all three approaches are combined [17]. Exercise has been most consistently beneficial in the treatment of the key components of frailty syndrome. The article published by Tsuchihashi et al. demonstrated that in-hospital exercise significantly improved frailty in patients with HCC [31]. Whereas the use of pharmacology as a treatment has not been adequately evaluated.

Our study has limitations, which should be considered when interpreting its data. Firstly, we used administrative codes (ICD codes), which are subject to misclassification. For example, classification and documentation of a condition, such as physical deconditioning may vary between different physicians and hospitals. ICD codes may not accurately represent the severity of illness and, therefore, may fail to include aspects including polypharmacy and the need for supportive care. Secondly, frailty was considered static at the time of admission. However, frailty should be regarded as a dynamic process that changes during hospitalization [32]. Therefore, in order to understand how frailty impacts mortality and outcomes in healthcare systems, further validation work is required. Thirdly, a population level database, such has the NIS, does not provide hepatic tumor stage or child classification.

Lastly, our cohort includes only hospitalized patients and does not include outpatient clinic visits. However, our study possesses several strengths that warrant merit. Our study used a large database, which is nationally representative of the US population. The NIS is a validated database used in clinical and epidemiological research. Our study also utilized a validated and novel hospital frailty score to stratify HCC patients according to low versus medium to high risk which is clinically applicable.

## 5. Conclusions and Future Perspectives

We observed that 50% of our national cohort of HCC patients had a medium/high frailty score. Frail patients were more likely to experience in-hospital patient mortality, hepatic encephalopathy, higher cost, and longer lengths of stay. Future studies are needed to evaluate the impact of rehabilitation programs in reducing frailty risk and adverse outcomes in patients with HCC. Although there are some proposed interventions to reduce frailty, including nutrition and exercise, these data are inconclusive. As the world’s elderly population increases, challenges posed by frailty will become a priority in most health care settings. Employing the hospital frailty score will allow for goal-oriented care for frail HCC patients as well as improving utilization of health care services.

## Figures and Tables

**Table 1:** Baseline characteristics.

Admission Characteristics	Low Frailty (*n* = 4648)	Medium/High Frailty (*n* = 6335)	*p*-Value
Age (years)			
<40	357	188	
40–64	2775	3149	<0.01
>65	1516	2998	
Ethinicy			
Caucasian	2414	3571	<0.01
African–American	655	957	
Hispanic	834	899	
Asian or Pacific Islander	341	389	
Native American	26	52	
Other	191	196	
Female	1176	1810	<0.01
Elective	1282	696	<0.01
Bed size			
Small	544	921	
Medium	1149	1612	<0.01
Large	2955	3802	
Teaching Status			
Rural	188	313	
Urban	720	1194	<0.01
Urban Teaching	3740	4828	
Payer Status			
Medicare	1782	3438	<0.01
Medicaid	1057	1132	
Private	1387	1370	
Self-Pay	183	200	
No Charge	22	16	
Other	196	170	
Length of Stay			
<7 days	3915	3985	<0.01
>7 days	733	2350	
Alcohol	882	1335	<0.01
Transplant	39	51	0.84
Hepatic Encephalopathy	52	188	<0.01
Depression	378	651	<0.01
Obese	276	597	<0.01

**Table 2:** Logistic regression model showing the effect of frailty on patient mortality.

Characteristics	*p*-Value	Odds Ratio	95% CI	
			Lower	Upper
Age (years)				
<40	Reference			
40–64	<0.001	4.885	2.395	9.963
>65	<0.001	5.049	2.441	10.444
Ethnicity				
Caucasian	Reference			
African–American	0.001	1.375	1.140	1.658
Hispanic	0.082	1.190	0.978	1.448
Asian or Pacific Islander	0.069	1.280	0.981	1.669
Native American	0.734	1.140	0.536	2.426
Other	0.434	1.161	0.799	1.688
Gender				
Male	Reference			
Female	0.375	0.931	0.796	1.090
Elective Admission	0.001	0.687	0.546	0.864
Bed size				
Small	Reference			
Medium	0.088	0.827	0.665	1.029
Large	0.018	0.791	0.652	0.960
Hospital Teaching Status				
Rural	Reference			
Urban	0.003	0.630	0.467	0.850
Urban Teaching	<0.001	0.455	0.345	0.601
Insurance Status				
Medicare	Reference			
Medicaid	0.134	1.181	0.950	1.469
Private	0.008	1.308	1.074	1.594
Self-Pay	0.018	1.543	1.077	2.212
No Charge	0.831	1.143	0.335	3.903
Other	<0.001	2.014	1.412	2.872
Length of Stay				
<7 days	Reference			
>7 days	0.099	1.130	0.977	1.307
Frailty				
Low frailty	Reference			
Medium/high frailty	<0.001	4.518	3.743	5.455
Alcohol	0.179	0.887	0.745	1.057
Transplant	0.255	0.551	0.197	1.539
Depression	<0.001	0.555	0.420	0.734
Obese	0.001	0.630	0.475	0.836

**Table 3:** Logistic regression model showing the effect of frailty on the risk of hepatic encephalopathy.

Characteristics	*p*-Value	Odds Ratio	95% CI	
			Lower	Upper
Age (years)				
<40	Reference			
40–64	0.027	4.880	1.201	19.830
>65	0.018	5.430	1.333	22.111
Length of Stay				
<7 days	Reference			
>7 days	<0.001	1.653	1.268	2.155
Frailty				
Low frailty	Reference			
Medium/high frailty	<0.001	2.256	1.640	3.104

**Table 4:** Adjusted Cox regression model showing the impact of frailty on the risk of in-patient mortality.

Characteristics	*p*-Value	Hazard Ratio	95% CI	
			Lower	Upper
Age (years)				
<40	Reference			
40–64	<0.001	5.519	2.733	11.146
>65	<0.001	5.668	2.769	11.606
Ethnicity				
Caucasian	Reference			
African–American	0.005	1.276	1.075	1.516
Hispanic	0.078	1.177	0.982	1.412
Asian or Pacific Islander	0.214	1.170	0.913	1.498
Native American	0.692	0.868	0.431	1.748
Other	0.871	0.972	0.685	1.378
Gender				
Male	Reference			
Female	0.083	0.878	0.757	1.017
Elective Admission	0.002	0.704	0.566	0.875
Bed size				
Small	Reference			
Medium	0.053	0.820	0.670	1.002
Large	<0.001	0.708	0.592	0.846
Hospital Teaching Status				
Rural	Reference			
Urban	<0.001	0.542	0.413	0.710
Urban Teaching	<0.001	0.353	0.275	0.453
Insurance Status				
Medicare	Reference			
Medicaid	0.576	1.059	0.866	1.296
Private	0.056	1.197	0.996	1.440
Self-Pay	0.034	1.432	1.028	1.994
No Charge	0.458	1.542	0.492	4.835
Other	0.002	1.681	1.217	2.322
Hepatic encephalopathy	<0.001	2.226	1.745	2.840
Frailty				
Low frailty	Reference			
Medium/high frailty	0.000	2.324	1.937	2.789
Transplant	0.344	0.622	0.232	1.665
Alcohol	0.248	0.909	0.773	1.069
Depression	<0.001	0.559	0.428	0.731
Obese	<0.001	0.592	0.452	0.773

## Data Availability

The data presented in this study are openly available in National Inpatient Sample using ICD-9 code 155.0.
